# Global Crystallographic Texture of Freshwater Bivalve Mollusks of the Unionidae Family from Eastern Europe Studied by Neutron Diffraction

**DOI:** 10.3390/life12050730

**Published:** 2022-05-13

**Authors:** Alexey Pakhnevich, Dmitry Nikolayev, Tatiana Lychagina, Maria Balasoiu, Orhan Ibram

**Affiliations:** 1 Borissiak Paleontological Institute, Russian Academy of Sciences, 117647 Moscow, Russia; alvpb@mail.ru; 2 Frank Laboratory of Neutron Physics, Joint Institute for Nuclear Research, 141980 Dubna, Russia; dmitry@nf.jinr.ru (D.N.); masha.balasoiu@gmail.com (M.B.); 3 Horia Hulubei National Institute for R&D in Physics and Nuclear Engineering, 077125 Bucharest, Romania; 4 Danube Delta National Institute for Research and Development, 820112 Tulcea, Romania

**Keywords:** crystallographic texture, pole figures, neutron diffraction, freshwater bivalve mollusk shells, Unionidae family

## Abstract

The crystallographic texture of the whole valves of bivalve mollusks from the family Unionidae *Unio pictorum* Linnaeus, 1758 and *Anodonta cygnea* Linnaeus, 1758 is studied using pole figures measured using neutron diffraction. The use of neutron diffraction, in contrast to X-rays, makes it possible to study the valves without destroying them. Thus, we can discuss the study of the global texture of the entire valve. It was revealed that the pole figures of aragonite in the valves repeat their shape. The pole density maxima for *U. pictorum* from the Danube Delta and the Gulf of Finland in the Baltic Sea, living at different salinities and temperatures, differs by 0.41 mrd. The maximum value of the crystallographic texture for *A. cygnea* from the Danube Delta was also measured (5.07 mrd). In terms of texture sharpness, it surpasses the shell of marine bivalve mollusks, which are partially or completely composed of aragonite. Although *U. pictorum* and *Mya arenaria* Linnaeus, 1758 have different microstructures, their pole figures are very similar in isolines pattern, but differ in pole density maxima. No relationship was found between the crystallographic texture and the microstructure in *U. pictorum*. In addition, we report good qualitative agreement between aragonite X-ray pole figures of *Sinanodonta woodiana* Lea, 1834 from the Czech river Luznice, and neutron pole figures of *U. pictorum* from the Danube Delta.

## 1. Introduction

Bivalve mollusks of the family Unionidae are the dominant freshwater Eurasian bivalve mollusks. They inhabit water bodies with both flowing (rivers, streams) and stagnant water (oligotrophic and eutrophic lakes and ponds) [1]. Unionidae are present in sea areas with low salinity, such as the Gulf of Finland in the Baltic Sea. These mollusks are an important component of human economic activity. They are a source of nacre and are used as food for agricultural livestock. Their larvae, glochidia, parasitize the gills and fins of fish, including commercial species. However, their fundamental ecosystem service is acting as a natural biofilter of freshwater bodies. *Unio pictorum* Linnaeus, 1758 filters fresh water at a rate of 550 mL/h; *Anodonta cygnea* Linnaeus, 1758 filters up to 400 mL/h [2]. Despite the fact that these mollusks live in fresh water, they grow large shells up to 130 mm long [1]. The microstructural elements of Unionidae shells are composed of three layers. Outside, they are covered with an organic conchiolin layer. Layers of mineral matter are located under it. The main chemical compound of this family of the mollusks’ shells is calcium carbonate as aragonite mineral. Unionid shells are 98% aragonite [1]. Aragonite crystals are grouped into two different microstructural components: prisms and plates. The prisms are oriented perpendicularly to the surface of the valves; the lamellar elements, the plates forming the nacre layer, are parallel to the same surface.

Relatively recently, crystallographic texture has been used to characterize mollusk shells. Crystallographic texture is a collection of orientations of a polycrystalline sample. It is formed during sample growth or formation, and influences the anisotropy of the sample’s physical properties. The shells are usually composed of calcite and aragonite crystallites which have trigonal and orthorhombic symmetry, respectively. These crystallites have anisotropic thermal and mechanical properties. The anisotropy of individual crystallites will cause anisotropy of a polycrystalline sample if the crystallographic texture is not uniform. The need to characterize crystallographic textures has been recognized primarily in materials science; quantitative texture analysis has been developed to a sophisticated level [3,4,5,6,7,8,9,10,11,12]. Quantitative information about crystallographic texture is contained in measured pole figures that are two dimensional distributions of relative volumes for specific crystallographic directions on a unit sphere. Values on a pole figure are given in mrd (multiple random distribution) units. The value 1 corresponds to uniform isotropic distribution of crystalline orientations.

Most shell pole figure measurements are carried out using X-rays or electron backscatter diffraction (EBSD) [13,14,15,16,17,18]. These techniques are very local, i.e., they allow measurement of only a small portion of a valve, and do not provide a complete picture of the distribution of crystal orientations in a shell. Due to the large neutron penetration depth, this type of diffraction makes it possible to measure the entire shell.

The aim of this work is to reveal the features of the global crystallographic texture of the valves of some representative bivalve mollusks of the Unionidae family, to establish whether they change depending on the temperature and salinity of the water, and to compare results of texture measurement using neutrons and X-rays.

## 2. Materials and Methods

### 2.1. Climatic Conditions and Salinity in the Habitats of the Studied Mollusks

**Figure 1:**
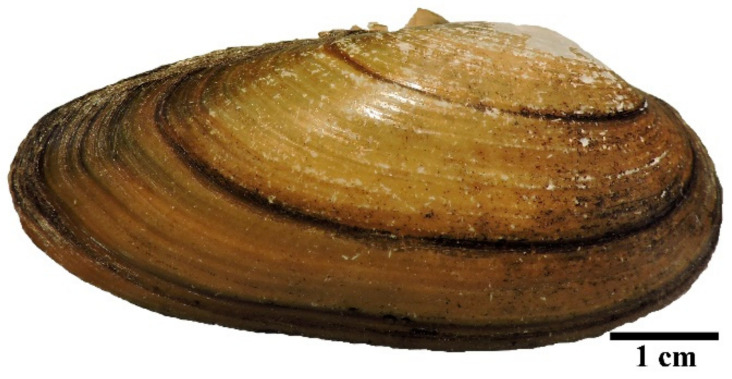
*Unio pictorum* Linnaeus, 1758 from the Danube Delta.

The valves of *U. pictorum* and *A. cygnea* bivalves collected from the Danube Delta, Tulcea area (Romania) were used in this study. For comparison, the valves of *U. pictorum* were taken from the coast of the Gulf of Finland in the Baltic Sea, near the Peterhof pier (St. Petersburg, Russia) (Figure 1).

The Danube River and the Gulf of Finland in the Baltic Sea differ significantly in terms of habitat. The salinity of these water basins varies; in fresh water bodies, it is 0.5‰ [19], and in the Gulf of Finland, salinity does not exceed 2‰ [20]. This desalination also promotes the spread of freshwater fauna in the bay, including Unionidae bivalve mollusks. The climates of both Tulcea and St. Petersburg are characterized as moderate, but their average temperatures differ. In the summer St. Petersburg is located slightly south of the 16 °C isotherm, and Tulcea is south of the 20 °C isotherm (according to July air temperatures). In winter St. Petersburg is on the −8 °C isotherm, while Tulcea is on the −4 °C isotherm (according to January air temperature values) [21]. In this regard, the living conditions of these mollusks differ.

It has been previously suggested [17,18,22] that the crystallographic texture of mussel shells *Mytilus edulis* Linnaeus, 1758 and *Mytilus galloprovincialis* Lamarck, 1819 is affected by sea acidification due to human activity, which results in increasing carbon dioxide content in the atmosphere and hydrosphere. Mussels were collected and cultured at different concentrations (partial pressures) of CO_2_, pH, and temperatures. The microstructure of the valves was analyzed using a scanning electron microscope and the crystallographic texture was analyzed using the EBSD method. Unfortunately, the studies did not explicitly describe from which part of the shell the investigated samples were cut. The authors of [17] indicate that the analysis was performed at or near the edge of the valve (commissure). However, none of the studies [17,18,22] clearly identify which valve sections are compared and depicted in figures for the microstructure and the crystallographic texture. The authors of [17] note that strong cold seeps of carbon dioxide were present in the water area of Ischia (Italy). Other gases were contained in insignificant amounts. Nevertheless, these conditions can be considered quite pessimal; they are unlikely to be present in other seacoast locations. In addition, a factor as important to marine organisms as salinity was not always taken into account. While salinity is almost the same in the habitat of *M. edulis* off the coast of China in Jiaozhou Bay and marine experimental aquariums in Japan [22], in Scottish experimental reservoirs (tanks) it varies from 28 to 37‰ [18].

In [23] it was revealed that an increase in the content of some chemical elements in sea water does not affect *M. galloprovincialis* crystallographic texture. The crystallographic texture in [23] was studied using neutron diffraction, and the content of elements was studied using neutron activation analysis. First attempts to study the influence of time on the global crystallographic texture of bivalve shells were made in [24]. This work shows that the sharpness of the crystallographic texture of calcite and aragonite has changed over 30 thousand years when comparing recent and fossil (Late Pleistocene) *M. galloprovincialis* and *Ostrea edulis* Linnaeus, 1758. For example, the sharpness of the crystallographic texture of calcite in mussel shells increased, while in oysters it decreased slightly. The sharpness of the aragonite texture in the shells of the mussel *M. galloprovincialis* also slightly increased. However, the factors that influenced these processes have not been established.

It has been established that crystallographic texture can be affected by various factors. In this work we are interested in comparing the texture of recent mollusk shells, of the same species or closely related, living in different conditions outside pessimal habitats, and analyzing the entire volume of several valves.

### 2.2. Crystallographic Texture Analysis of Shells’ Minerals

We use time-of-flight neutron diffraction to characterize crystallographic texture. Penetration depth of the neutrons in calcite is approximately several centimeters. The reason to use neutron diffraction for obtaining primary information about crystallographic texture is connected with a sample gauge volume. This technique characterizes the entirety of the valves. In the case of X-ray diffraction, the penetration depth is approximately 100 µm; the EBSD technique provides information for only a few atomic layers. X-ray and EBSD can characterize the sample only locally; when using neutrons, texture information is extracted not only from the surface of the sample, but also from the depth. Thus, neutron diffraction is proving to be a powerful tool for studying global texture in centimeter-sized samples. The dimensions of the gauge volume of a sample in a neutron experiment are determined using the cross section of the neutron beam and the dimensions of the detector, on the one hand, and using the features of the interaction of the material under study with neutrons, on the other. The measurement time depends on the neutron flux density of the sample; the scattering power of the studied material; the intensity of the measured Bragg reflection; and the texture sharpness. The condition of Bragg diffraction at a fixed scattering angle can be fulfilled simultaneously for different planes using a polychromatic neutron beam which is implemented in the time-of-flight technique. All of the pole figures can be measured simultaneously using this technique.

**Figure 2:**
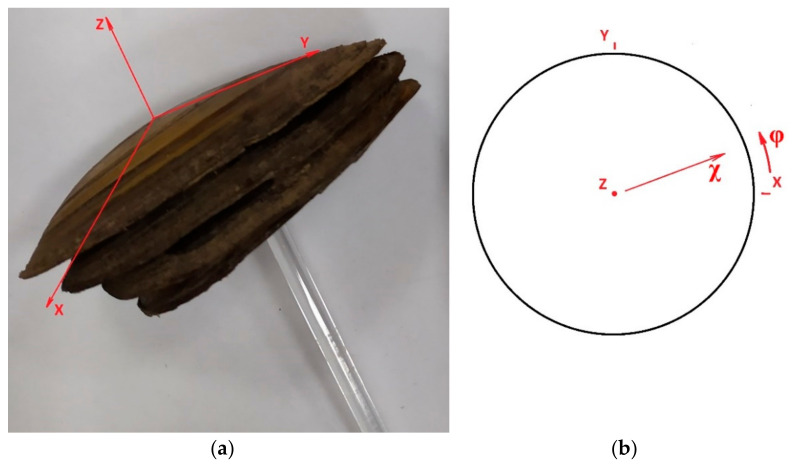
(**a**) Global coordinate system associated with the entire shell, (**b**) stereographic projection of the coordinate system. φ is azimuth angle and χ is tilt angle, which determines the pole position on the stereographic projection.

The experimentally measured pole figures are associated with the coordinate system of the measured sample. In the case of X-ray or EBSD, the specimen is a small piece of the valve, which is not easily related to the coordinate system of the entire valve. This can be accomplished by studying the entire shell, which is possible using neutron scattering. The global coordinate system connecting the entire shell in the neutron experiment, as well as its stereographic projection, are shown in Figure 2. The pole figures are usually collected by varying the angle of rotation φ that measures the azimuth of the sample in the surface plane, and the tilt angle χ that defines the amount of tilting of the sample with respect to the normal, to the sample surface. φ and χ are varied from 0–360° and 0–90°, respectively, as shown in Figure 2b.

Another disadvantage of the X-ray texture measurement method is the possibility of measuring only incomplete pole figures. The spatial orientation of the sample when measuring pole figures using the X-ray method in the reflection mode is changed by rotating around two mutually perpendicular planes. The inclination of the sample relative to the incident X-ray beam leads to a change in the area of the irradiated surface and the relative path of the incident and reflected beams in the sample. This leads to a change in the recorded intensity of the reflected beam. Therefore, absorption corrections are introduced for different tilt angles. At small angles of incidence of the beam on the sample surface the focusing condition is violated; the “defocusing effect” appears, which manifests itself in an additional drop in intensity as a result of increasing the tilt angle, and the correction for absorption no longer provides a complete correction. Therefore, the corrections of intensity for defocusing are introduced, which are measured on reference textureless samples or using calculations. In addition, the defocusing effect leads to broadening of the diffraction lines. Moreover, the broadening increases with increasing inclination of the sample and, starting at angles of 70°, the corrections become insufficient.

To obtain complete pole figures using X-ray texture analysis, it is necessary to calculate them using the orientation distribution function (ODF) reconstructed from the measured incomplete pole figures [5]. In the neutron texture experiment, a direct measurement of complete pole figures can be realized, since the transmission scheme is possible.

The neutron pole figures presented in this work were obtained at the Frank Laboratory of Neutron Physics of the Joint Institute for Nuclear Research (Dubna). We used the SKAT (Spectrometer for Quantitative Texture Analysis) setup located on channel 7A-2 of the IBR-2 pulsed nuclear reactor [25]. Time-of-flight neutron diffraction was used to measure diffraction reflections corresponding to the minerals of which the shells are composed. The pole figures corresponding to the most intense aragonite diffraction reflections (012)/(121) and (040)/(221) were extracted from diffraction patterns.

The SKAT instrument consists of a detector ring (diameter 2 m) with 19 detector–collimator complexes which are located at the same scattering angle 2θ = 90°. The detector modules are mounted around the circumference of the detector ring in such a way that the step on the pole figure is 5°. The sample rotates with a step of 5° around the horizontal axis which has an angle of 45° with respect to the incident neutron beam. Rotation is carried out using a goniometer. Thus, for each complete pole figure, 19 × 72 = 1368 diffraction patterns are recorded. Thanks to the time-of-flight technique, the pole figures of all minerals (phases) present in the sample are measured simultaneously, i.e., they are extracted from the same diffraction patterns. It should be noted that each individual pattern is measured under the same conditions, which means that pole figures obtained in this way do not need to be corrected. Because of the large depth of neutron penetration, centimeter-sized bulk samples can be easily examined in transmission geometry. The neutron beam cross section of 50 × 90 mm makes it possible to measure large samples. In order to collect a volume of material sufficient for measurements, 3–4 valves were glued one after the other with a special two-component adhesive. Either only the left, or only the right, valves were used. Then, the prepared sample was mounted on a glass pin and fixed in the goniometer of the setup. The measurement time for each sample was 22 h. Another advantage of neutron diffraction experiments is the absence of additional requirements for surface treatment and sample shape, which are imposed in X-ray texture studies.

**Figure 3:**
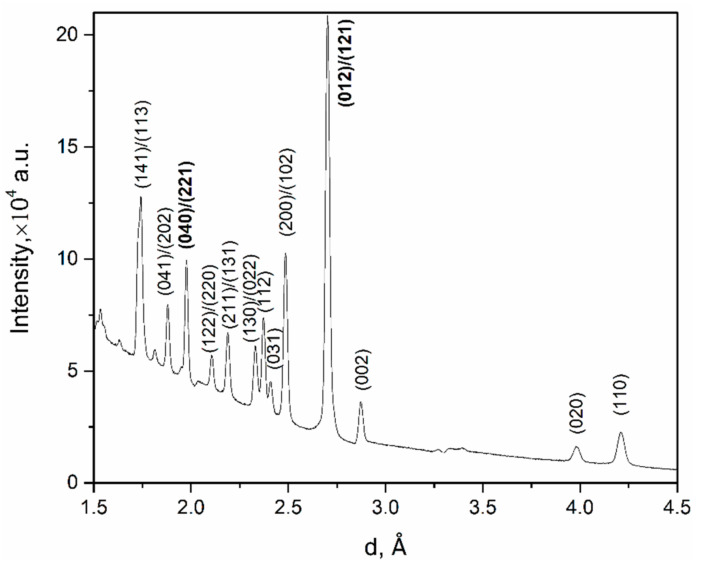
Neutron diffraction pattern obtained by summing 1368 partial diffractograms for *Unio pictorum* valves from the bank of the Danube River. The most intense diffraction reflections for which pole figures have been extracted are highlighted in bold type.

The most intense diffraction reflections in each of the recorded patterns were analyzed using the Pole Figure Extractor program [26] to determine the orientation distribution of the corresponding crystallographic planes in the valves. The intensity of one reflection corresponding to the crystallographic plane with certain Miller indices shows one point on the pole figure indicated by these indices. All pole figures were normalized and smoothed with the same parameter. Figure 3 shows a neutron diffraction pattern obtained by summing 1368 partial diffractograms for *U. pictorum* valves from the bank of the Danube River. The pole figures for the most intense diffraction reflections corresponding to the crystallographic planes with Miller indices (012)/(121) and (040)/(221) for aragonite were extracted from the neutron patterns.

The more ordered the mineral crystals, the higher the intensity of the pole figure (pole density), expressed in units of isotropic distribution (multiple random distribution, mrd). An increase in the intensity of the pole figure is interpreted as an intensification of the texture. A pole density value of 1 means that the orientations of corresponding crystallographic planes are uniformly distributed in all directions in the sample. Regions of pole figures with densities greater than 1 indicate that more lattice planes are aligned in those directions than in a random sample, and vice versa.

The microstructure of the valve of the mollusk *U. pictorum* was studied using a scanning electron microscope (SEM) TESCAN//VEGA2 (Borissiak Paleontological Institute, RAS, Moscow, Russia). The survey was carried out without spraying, in a low vacuum, on a shell chip.

## 3. Results

**Figure 4:**
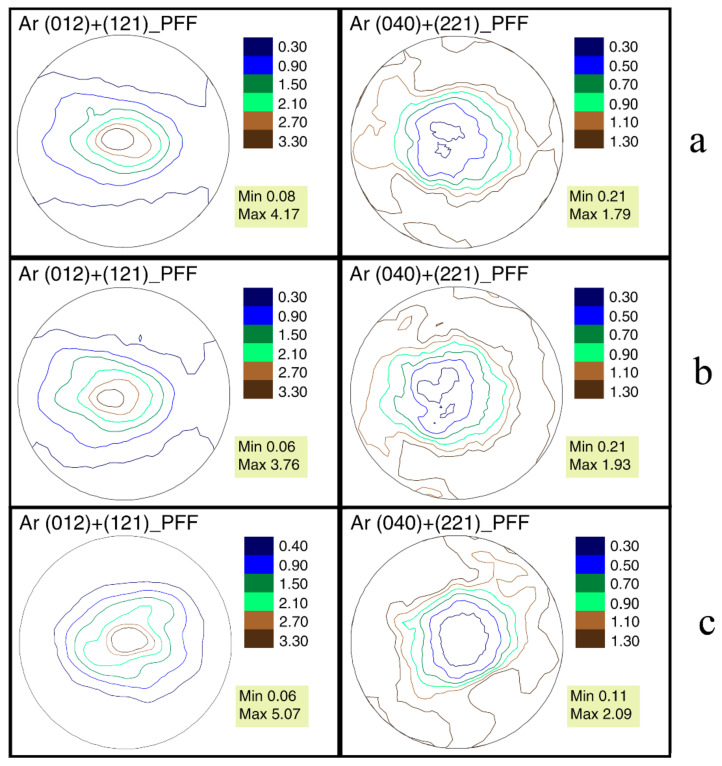
Neutron pole figures of aragonite in the shells of bivalve mollusks: (**a**) *Unio pictorum* from the Gulf of Finland; (**b**) *Unio pictorum* from the Danube Delta; (**c**) *Anodonta cygnea* from the Danube Delta.

**Figure 5:**
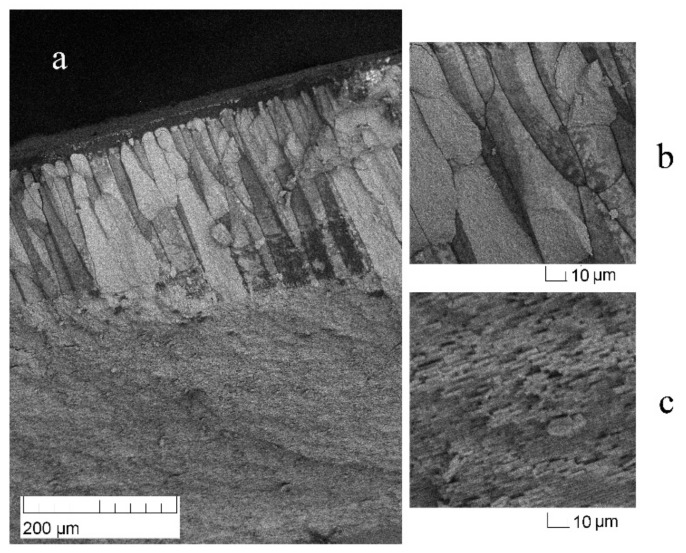
Microstructure of *Unio pictorum* valve from the Gulf of Finland, scanning electron microscope images: (**a**) general view of the chip, (**b**) porcelain layer, and (**c**) pearlescent layer.

In all studied valves, only aragonite (CaCO_3_) was found. For each of the collected samples, neutron pole figures were obtained. For the crystallographic direction (012)/(121), it is noticeable that the crystals are oriented in a wide range. The contours of the pole figures are elongated horizontally, i.e., the aragonite crystals repeat the shape of the shell (Figure 4). The SEM image (Figure 5) shows that the crystals are organized into various microstructural units such as prisms and plates, positioned at different angles to the shell surface. As a result, one can expect several sharp peaks on the pole figures. However, the different arrangement of microstructural elements relative to the surface of the mollusk’s shells does not affect the measured texture in any way. That is, the microstructure and texture of the shell are weakly related. Similar conclusions for different mollusk species were reported in [27].

The appearance of the *U. pictorum* pole figures from Tulcea and Peterhof is very similar for *A. cygnea*. The sharpness of the crystallographic texture of the valves of *U. pictorum* from the Danube delta is 4.17 mrd, and from the Gulf of Finland 3.76 mrd, respectively. We estimate the texture sharpness using the maxima of the aragonite pole figure (012)/(121). The difference in texture sharpness of the same species in different habitat conditions is 0.41 mrd. This is a very important value, since there are almost no data regarding the difference in crystallographic texture in organisms living in different conditions.

The isolines of the pole figures of *A. cygnea* are also horizontally elongated. However, the pole figure is more rounded than that of the *U. pictorum*. Aragonite crystals are also oriented in the shape of the valves. An important feature of the shell texture of this species is its sharpness of 5.07 mrd. Here, bivalve, whose shells also include aragonite, should be compared. The inner layers of mussel shells are composed of nacre. Earlier we studied [25] the crystallographic texture of the shells of three species of the genus *Mytilus* Linnaeus, 1758. The arrangement of their aragonite crystals is also similar to the shape of the valves. However, the sharpness of the aragonite pole figures does not exceed 3.36 mrd for *M. edulis*.

Some shells of marine bivalve mollusks are composed entirely of aragonite and have a microstructure different from that of the shells of the freshwater mollusks studied. It was interesting to compare the pole figures of *U. pictorum* and a similar marine mollusk. The valves of *Mya arenaria* Linnaeus, 1758 from the Kazantip Bay in the Sea of Azov (Russia) were used for comparison [25]. The external pole figures of *My. arenaria* are very similar to those of *U. pictorum;* however, the sharpness of its pole figure (012)/(121) is 2.63 mrd, which is less than that of all the freshwater mollusks we studied. It was possible to once again confirm, as was concluded in [27], that crystallographic texture is weakly related to microstructure, because the aragonite pole figures of *U. pictorum* and *My. arenaria* are qualitatively similar despite the significant microstructure difference.

## 4. Discussion

To be able to compare the neutron pole figures with X-ray pole figures obtained in the recent study [28] for *Sinanodonta woodiana* Lea, 1834 shells, the orientation distribution function (ODF) was calculated. This was accomplished using the MTEX program [29] choosing two pole figures, (012)/(121) and (040)/(221), measured using neutron diffraction for *U. pictorum* from the Danube Delta.

ODF is a three-dimensional probability density function, which is the relative volume of grains with a particular orientation, expressed, for example, as Euler angles. It can be calculated using measured pole figures. ODF contains complete information about crystallographic texture, and can therefore be used to recalculate pole figures that could not be extracted from diffraction patterns. In addition, ODF can be used to calculate complete pole figures from incomplete X-ray measured pole figures. These things considered, ODF makes the calculation of macroscopic properties of a sample using the corresponding properties of the microscopic constituents possible [3,9,10,11].

**Figure 6:**
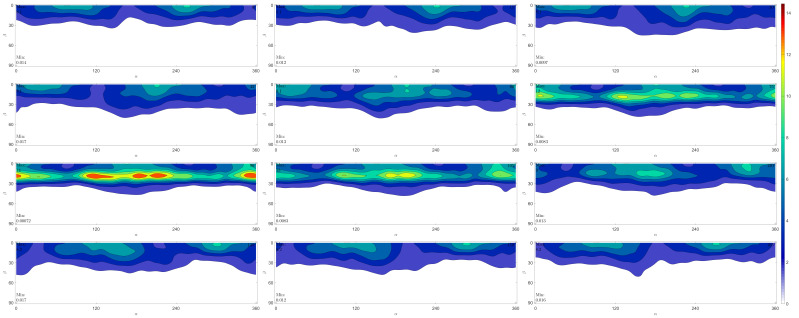
Orientation distribution function of *Unio pictorum* from the Danube Delta (15° γ-sections, Matthies’s notation).

*Sinanodonta woodiana* is also a freshwater bivalve mollusk whose shell is composed entirely of aragonite. It was collected from the Czech river Luznice. The ODF calculated for the sample of *U. pictorum* from the Danube Delta is shown in Figure 6 in the form of a section along the Euler angle γ in Matthies notation [5].

**Figure 7:**
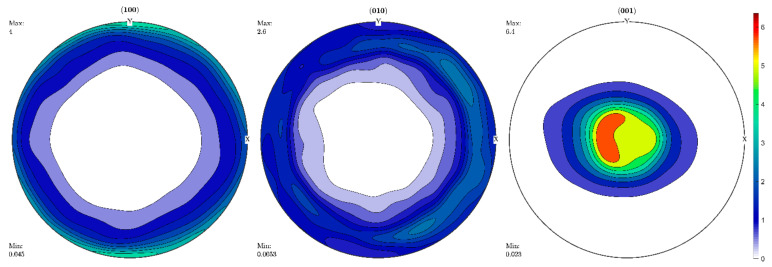
Pole figures for *Unio pictorum* from the Danube Delta recalculated from the orientation distribution function presented in Figure 6.

The pole figures (100), (010), (001) were calculated based on the reconstructed ODF (Figure 7), as they were in [28], for the measured incomplete X-ray pole figures.

There is a good qualitative agreement between the aragonite X-ray pole figures of *S. woodiana* and the neutron pole figures of *U. pictorum*. The best external similarity is observed in pole figures (001). The maximum values of the pole density, which characterize the sharpness of the texture, practically coincide for the (010) and (100) pole figures, while the least similar match is observed for the (001) pole figure. The conclusion made for *S. woodiana* that the c-axis of aragonite (the normal to the crystallographic plane (001)) is perpendicular to the inner surface of the shell is also valid for *U. pictorum*.

## 5. Conclusions

According to the results gathered in the current study the pole figures (012)/(121) of aragonite in the valves of the mollusks *U. pictorum*, living at different temperatures and salinity, are very similar in their isolines patterns, but differ in their sharpness by 0.41 mrd. The sharpest aragonite texture observed for *A. cygnea* is 5.07 mrd according to pole figure (012)/(121). The distribution of aragonite crystals in the valves reflects their external shape. Although *U. pictorum* and *My. arenaria* have different microstructures, their pole figures are very similar in isolines pattern, but differ in pole density maxima. It has been established that the shell microstructure is weakly related to its crystallographic texture. The pole figures recalculated using the ODF reconstructed from aragonite neutron pole figures for *U. pictorum* from the Danube Delta are qualitatively very similar to those measured using X-rays for *S. woodiana* from the Czech river Luznice.

## Data Availability

Not applicable.
